# An overiew of non medical prescribing across one strategic health authority: a questionnaire survey

**DOI:** 10.1186/1472-6963-12-138

**Published:** 2012-06-01

**Authors:** Molly Courtenay, Nicola Carey, Karen Stenner

**Affiliations:** 1 Division of Health and Social Care, University of Surrey, Guildford, Surrey, UK

**Keywords:** Non-medical prescribing, Independent prescribing, Supplementary prescribing, Community practitioner prescribers, Survey, Medicines management

## Abstract

**Background:**

Over 50,000 non-medical healthcare professionals across the United Kingdom now have prescribing capabilities. However, there is no evidence available with regards to the extent to which non-medical prescribing (NMP) has been implemented within organisations across a strategic health authority (SHA). The aim of the study was to provide an overview of NMP across one SHA.

**Methods:**

NMP leads across one SHA were asked to supply the email addresses of NMPs within their organisation. One thousand five hundred and eighty five NMPs were contacted and invited to complete an on-line descriptive questionnaire survey, 883 (55.7%) participants responded. Data was collected between November 2010 and February 2011.

**Results:**

The majority of NMPs were based in primary care and worked in a team of 2 or more. Nurse independent supplementary prescribers were the largest group (590 or 68.6%) compared to community practitioner prescribers (198 or 22.4%), pharmacist independent supplementary prescribers (35 or 4%), and allied health professionals and optometrist independent and/or supplementary prescribers (8 or 0.9%). Nearly all (over 90%) of nurse independent supplementary prescribers prescribed medicines. Approximately a third of pharmacist independent supplementary prescribers, allied health professionals, and community practitioner prescribers did not prescribe. Clinical governance procedures were largely in place, although fewer procedures were reported by community practitioner prescribers. General practice nurses prescribed the most items. Factors affecting prescribing practice were: employer, the level of experience prior to becoming a non-medical prescriber, existence of governance procedures and support for the prescribing role (p < 0.001).

**Conclusion:**

NMP in this strategic health authority reflects national development of this relatively new role in that the majority of non-medical prescribers were nurses based in primary care, with fewer pharmacist and allied health professional prescribers. This workforce is contributing to medicines management activities in a range of care settings. If non-medical prescibers are to maximise their contribution, robust governance and support from healthcare organisations is essential. The continued use of supplementary prescribing is questionable if maximum efficiency is sought. These are important points that need to be considered by those responsible for developing non-medical prescribing in the United Kingdom and other countries around the world.

## Background

Increasing socioeconomic and political demands on United Kingdom (UK) healthcare systems have seen the extension of prescribing rights to groups of non-medical healthcare professionals. Enhancing the role of these healthcare professionals to include prescribing is fundamental to improvements in the quality and accessibility of healthcare [1]. Although several countries (e.g. Australia, Ireland, and the United States), have implemented prescribing by non-medical healthcare professionals and, it is planned for in others (for example the Netherlands) [2,3], no other country has such extended non-medical prescribing (NMP) rights as the UK.

Community nurse practitioners in the UK were the first group to be provided with the capacity to prescribe, and these community practitioner prescribers are able to independently prescribe from a limited list of medicines and conditions (including minor ailments and wound dressings), listed in the Nurse Prescribers Formulary for Community Practitioners [4]. Independent prescribing rights were extended in 2001 to include other groups of registered nurses [5]. Nurse independent supplementary prescribers (NISPs) are able to independently prescribe any medicine (including controlled drugs and unlicensed medicines) [6] and can also prescribe any medicine as a supplementary prescriber [1]. Supplementary prescribing, which takes place after assessment and diagnosis of a patient’s condition by a doctor, involves the development of a Clinical Management Plan (agreed by the patient, doctor and supplementary prescriber) which outlines the list of medicines from which the supplementary prescriber is able to prescribe for a patient [5].

Pharmacists were given supplementary prescribing rights in 2003 and later legislative changes also enabled this group the same independent prescribing rights as nurses [7]. More recently optometrists, and allied health professionals (AHPs) (i.e. physiotherapists, radiographers, and chiropodists/podiatrists) have been able to train as supplementary prescribers and optometrists are now able to prescribe independently [8]. Training to become a NMP prescriber typically involves 27 days in the classroom and 12 days in practice under the supervision of a doctor [4].

There are approximately 33,000 community practitioner prescribers, 23,000 NISPs, 2000 pharmacist independent supplementary prescribers (PISPs), and several hundred AHPs and optometrist, working across the UK, with prescribing capability [9]. This represents between 1% to 3% of the current nursing, pharmacy, AHPs and optometrist workforce [10]. The numbers are set to rise with the extension of prescribing rights to other non-medical healthcare professional groups [11].

Stakeholders are generally satisfied with NMP [12-15] and report that it increases the accessibility and flexibility of services [16,17]. A number of benefits for NMPs themselves have also been reported including greater autonomy and increased job satisfaction, more time with patients and the ability to provide a complete episode of care, increased self-confidence, and time savings [18-21]. There are however, wide variations in the numbers of prescribers both within and across organisations [22] and barriers to NMP have been reported including restrictions of local arrangements (such as inability to access prescription pads), inability to computer generate prescriptions, lack of peer support, organisational and policy restrictions, and difficulties in fulfilling continuing professional development needs [23]. Inconsistencies in the clinical governance systems within which NMPs work have also been identified [15] and such inconsistencies can influence prescribing activity and its on-going use.

The profile and prescribing practices of NISPs [23] and the prescribing activity of nurse and pharmacist independent prescribers [24] have been explored in two national surveys. Additionally, a number of small studies have explored the impact and effectiveness of community practitioner prescribers [25]. However, there is no evidence available with regards to the extent to which NMP (including community practitioner prescribers, nurse, pharmacist and AHP independent/supplementary prescribers) has been implemented within healthcare organisations across a large geographical area. At the time of the study, the National Health Services (NHS) in England was divided into 10 areas and managed by strategic health authorities (SHAs). Each SHA had the responsibility to manage the local NHS across large geographical areas that encompass numerous health care organisations (including primary care trusts (PCTs), acute trusts, mental health trusts and general practices). The aim of the study was to provide an overview of NMP across one SHA. The specific objectives were to identify:

1) The non-medical healthcare professionals qualified to prescribe medicines i.e. their job title, the care setting in which they worked, and their clinical experience and qualifications

2) The mode of prescribing used by these healthcare professionals, the frequency with which they prescribe, and the different ways in which the prescribing qualification is used

3) The safety and clinical governance systems within which these healthcare professionals work

## Methods

### Design

An on-line descriptive questionnaire survey

### Participants

Eight hundred and eighty three NMPs within one SHA

### Questionnaire

SurveyMonkey–a tool for creating web surveys–was used to develop an on-line questionnaire (see Additional file 1). The questionnaire, informed by previous work undertaken by the researchers [15,23,26], was divided into 4 sections. Questions were mainly fixed choice with room for open ended comment. Section 1 collected general demographic information including job title, county in which the participant worked, employer, highest academic qualification, care setting and number of NMPs in the team. Section 2 asked questions specific to participants prescribing background including prescribing qualification held, number of years qualified as a prescriber, number of years’ experience in main area of prescribing practice prior to undertaking the prescribing programme, specialist training prior to becoming a prescriber. Section 3 comprised questions about prescribing practice. Questions included the method of prescribing currently used and the number of items prescribed, the different ways in which the prescribing qualification was used (i.e. participants were asked to indicate from a list of 12 statements the methods they used/did not use), and the therapy areas in which participants prescribed. The final section focused on clinical governance. Participants were asked to indicate from a list of 11 statements their experience of the clinical governance systems in place within their organisation. Participants were also asked whether or not they had received support from their NMP lead.

### Data collection

**Figure 1:**
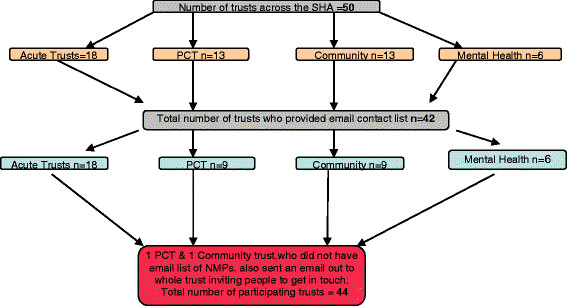
Number of Trusts across the Strategic Health Authority who provided email contact list.

Guidance [1] refers to the responsibilities of NHS organisations to develop a strategic plan for NMP. This plan includes the appointment of an NMP lead responsible for the implementation of NMP within an organisation. As part of safety and clinical governance arrangements, the NMP lead is responsible for the maintenance of a current database containing the details of NMPs within their organisation. Information supplied by the SHA, identified that 45 NMP leads were designated as responsible for NMPs within the 50 trusts across the 6 counties (Suffolk, Essex, Cambridgeshire, Norfolk, Hertfordshire, Bedfordshire) comprising the East of England (EoE) SHA (see Figure 1). Each of these leads were contacted by the researchers and asked if they would supply the email addresses of all NMPs listed on their database. In order to comply with SHA policy and the Data Protection Act (1998), an NHS laptop and an NHS email address was used for all email communication between a researcher (NC), NMP leads and NMPs.

Forty leads, responsible for 44 trusts, responded. Although 38 were able to provide a current electronic database of NMPs, two were unable to do so. These two leads emailed the NMPs for whom they were responsible, and requested that they made contact with the researchers in order to participate in the survey.

Two thousand and nine NMPs (comprising community practitioner prescibers, NISPs, PISPs, optometrists independent/supplementary prescribers, and AHP supplementary prescribers) were identified, of whom 1,869 had email addresses. An email containing an invitation letter, outlining the purpose of the study, and the link to the on-line questionnaire was sent to each NMP with an email address. Delivery receipts were requested. One thousand five hundred and eighty five emails were acknowledged as delivered. Participants were sent three follow-up reminder emails. Data collection took place between November 2010 and February 2011.

### Ethical consideration

Ethical approval for the study was provided by the University of Surrey. The study was deemed a service evaluation by Cambridgeshire 4 Research Ethics Committee.

### Data analysis

Microsoft Excel and SPSS version 17 were used for data entry and analysis. Descriptive statistics were used to describe the demographic nature of the sample. Analysis of variance (ANOVA) was used to explore whether the number of items prescribed differed according to individual demographic variables such as job title, employer, care setting, and time since qualifying as prescriber. General linear modelling (GLM), a popular generalisation of the linear regression model [27], was also used to explore whether demographic variables (i.e. job title, employer, care setting, and time since qualifying as prescriber) contributed significantly to explaining the variation in the ways the prescribing qualification was used, and the extent to which clinical governance procedures were in place. Chi-square was used to explore the difference between demographic variables and the level of support received before, during and after the prescribing programme. Content analysis was used to analyse free text comments.

## Results

Of the 1,585 participants invited to complete the survey, 883 (55.7%) participants responded.

### Demographic information

**Figure 2:**
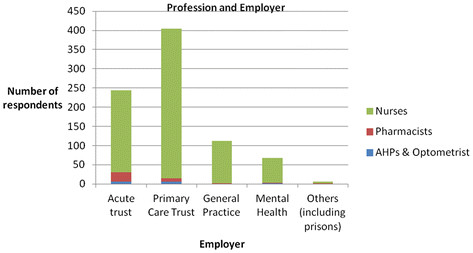
Profession and Employer.

The demographic data of the sample are presented in Table 1. Participants were from all six counties across the SHA, with 307 (34.8%) based in Essex. Of those who reported their job title, 826 (94.8%) respondents were nurses, the largest majority (n = 254 or 28.8%) of whom had specialist roles. Thirty six (4.1%) respondents were pharmacists, 9 (1.0%) were AHPs and this included one optometrist. The majority of nurses (n = 391, 47.3%) were employed by PCTs (including community trusts and provider services) whereas a higher percentage of pharmacists (n = 24, 68.6%) and AHPs (n = 7, 77.8%) were employed by acute trusts (see Figure 2). Degrees or higher degrees were held by 632 (71.5%) participants (see Table 1). The number of NMPs per team ranged from one (n = 278, 31%) to over 10 (n = 37, 4.3%) (mean =5.48, median = 2.0). Just over a third (n = 299, 33.9%) of respondents indicated that there were plans to increase these numbers.

**Table 1:** Demographic Details

	***n = number of responses***	**% of total sample**
**Job Title**		
**Specialist nurses** (clinical nurse specialists, specialist nurse practitioners, nurse clinician, paediatric specialist nurse)	254	28.8
**Community Nurses** (community matron, children’s community nurse, health visitor, district nurse, school nurse)	201	22.8
**General practice nurses** (practice nurses and nurse practitioners)	150	17.0
**Senior clinical nurses** (nurse consultant, lead nurse, ward manager, sister, charge nurse, team leader, modern matron)	120	13.6
**Mental Health Nurses**(community psychiatric nurse, primary care link worker, liaison nurse, clinical co-ordinator)	54	6.1
**Pharmacists** (team leader/manager, senior clinical pharmacist, senior pharmacist (care homes, elderly, transplant), education and training pharmacist, community pharmacist, practice support pharmacist)	36	4.1
**Nurse Manager**s (Director of nursing, service lead, information manager)	33	3.7
**Allied Health Professionals** (clinical specialist physiotherapist (chronic pain, elderly), podiatric diabetes specialist, clinic radiographer) &**Optometrist**	9	1.0
**Others nurses** (practice development, education, research)	14	1.6
**Geographical location**		
Essex	307	34.8
Norfolk	161	18.2
Cambridgeshire	142	16.1
Suffolk	119	13.5
Hertfordshire	76	8.6
Bedfordshire	65	7.4
**Employer**		
Primary care Trust (incl community trust and other provider services)	406	46.0
Acute Trust	276	31.3
General Practice	112	12.7
Mental Health	64	7.2
Others (including prisons)	8	0.9
**Care setting**		
Primary care (including intermediate care)	503	57.0
Secondary Care (including tertiary care)	240	27.2
Primary and Secondary Care	65	7.4
Mental Health (including learning disabilities, & prisons)	53	6.0
**Prescribing qualification**		
Nurse Independent Supplementary Prescriber(NISP)	590	66.8
Community Practitioner Prescriber (CP)	198	22.4
Pharmacist Independent Supplementary Prescriber & Pharmacist Supplementary Prescriber (PISP)	35	4.0
Other prescribing qualifications (Physiotherapist, Podiatrist or Radiographer Supplementary Prescriber, Optometrist Independent Supplementary Prescriber)	8	0.9
**Years qualified as a prescriber**		
< 1 year	50	5.7
1-3 years	257	29.1
3-5 years	223	25.3
> 5 years	287	32.5
**Experience in area of practice before becoming prescriber**		
< 1 year	93	10.5
1-2 years	43	4.9
2-5 years	153	17.3
> 5 years	522	59.1
**Highest level of educational attainment**		
Certificate	62	7.0
Diploma	156	17.7
Degree	441	49.9
Higher Degree (Masters or PhD)	191	21.6
**Level of specialist training before prescribing programme**		
Diploma module	74	8.4
Degree module	156	17.7
Masters module	44	5.0
Degree and/or masters module plus study days &/or other training	241	27.3
Accredited study days & other training (e.g. conference/drug company)	76	8.6
No specialist training	216	24.5

Percents do not add to 100% in each category as some participants did not complete every question.

### Prescribing background

Five hundred and ninety (66.8%) participants reported they were NISPs, nearly a quarter (n = 198, 22.4%) were community practitioners, with only small numbers (n = 43, 4.9%) of pharmacists, AHPs, or optometrist independent and/or supplementary prescribers (see Table 1). The majority of participants (n = 510, 57.8%) had been qualified to prescribe for more than three years and 675 (76.4%) indicated that they had more than two years’ experience in their area of practice before undertaking the prescribing programme. Four hundred and forty one (50%) reported they had undertaken degree and/or masters level specialist training in their area of prescribing practice.

### Prescribing practice

Five hundred and seventy eight (65.5%) participants reported that they currently used independent prescribing and 28 (3.2%) that they only used supplementary prescribing. A further 58 (6.6%) reported that they used both independent and supplementary prescribing. In addition to being ‘only qualified as a supplementary prescriber (n = 39) the most frequently cited reasons for using supplementary prescribing were ‘trust policy’ (n = 39), ‘personal preference’ (n = 26) and ‘controlled drug restrictions’ (n = 24) (Legislation restricting independent prescribing of controlled drugs by nurses and pharmacists was amended following data collection in this study [6].

One hundred and thirty three (15.1%) participants reported they did not currently prescribe. This included 59 (29.7%) of those who reported they had the community practitioner prescribing qualification, 56 (9.5%) of NISPs, 13 (37.1%) of PISPs and three (37.5%) AHPs (including an optometrist). Reasons for not prescribing identified from free text comments included role change (n = 56), procedural delays (e.g. lack of electronic prescribing and access to patient notes) (n = 27), formulary restrictions or trust policy (n = 26), a lack of support from employers and managers and lack of continuing professional development (n = 16). Community practitioners more often reported procedural delays (n = 23) and were the only group to mention a lack of continuing professional development and confidence as a reason for not prescribing. AHPs (n = 3) reported restrictions in the applicability of supplementary prescribing as the main reason for not prescribing.

Participants (n = 672, 76.1%) reported using independent prescribing to prescribe a mean number of 16.4 items per week and 254 (28.9%) reported using supplementary prescribing to prescribe a mean number of 5.7 items per week (see Table 2).

**Table 2:** Number of items prescribed by using independent and supplementary prescribing in a typical week

**Number of items per week**	**Independent prescribing**	**Supplementary prescribing**
0	69 (10.3%)	170 (66.9%)
1-5	219 (32.6%)	53(20.9%)
6-10	120(17.9%)	16 (6.3%)
11-20	85(12.6%)	8 (3.1%)
21-30	59 (8.8%)	4 (1.6%)
31-40	27 (4.0%)	1(0.4%)
41-50	24 (3.6%)	0 (0.0%)
>50	69 (10.3%)	2 (0.8%)
**Total number of respondents**	**672 (100%)**	**254 (100%)**

Using ANOVA it was evident that the number of items prescribed using independent prescribing was affected by the prescribing qualification. The mean number of items independently prescribed by NISPs (n = 484, mean =18.7), was significantly higher than PISPs, (n = 18, mean =12), or CPs, (n = 111, mean =7.2) (p < 0.001).

Additional analysis using ANOVA identified the number of items prescribed using independent prescribing was also significantly affected by job title, employer, care setting and time since qualifying (p < 0.001). General practice nurses, those employed in general practice, participants working across primary and secondary care and those with more than 5 years’ experience prior to undertaking the prescribing programme prescribed the greatest number of items each week. Those employed in general practice prescribed the greatest number of items per week (n = 103, mean = 38.9) and those employed by mental health trusts prescribed the lowest (n = 10, mean = 5.0).

Prescribing qualification, job title, employer, care setting and time since qualifying were not found to have any significant effect on the number of items prescribed using SP (p > 0.05).

### Therapy areas

**Figure 3:**
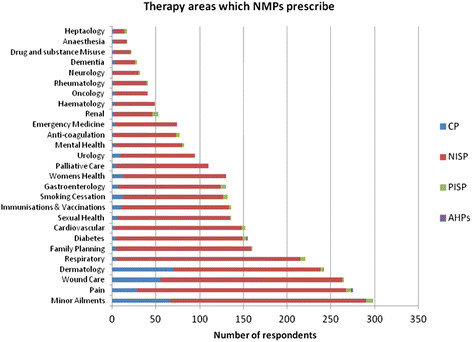
Therapy areas which NMPs prescribe.

The range of therapy areas for which participants prescribed are shown in Figure 3. Areas where the greatest number of NISPs prescribed were pain (239, 40.5%), minor ailments (n = 224, 40.0%) and respiratory (n = 210, 35.6%). Community practitioners prescribed most often for dermatology (n = 70, 35.5%), minor ailments (n = 66, 33.3%) and wound care (n = 55, 27.8%). In addition to minor ailments (n = 8, 22.9%), renal (n = 7, 20.0%) and respiratory (n = 6, 17.1%) were also therapy areas in which more PISPs prescribed.

### Ways in which the prescribing qualification is used

**Figure 4:**
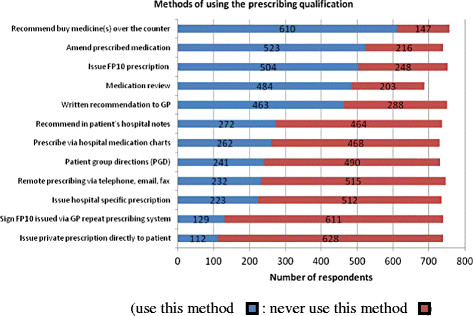
Methods of using the prescribing qualification.

**Figure 5:**
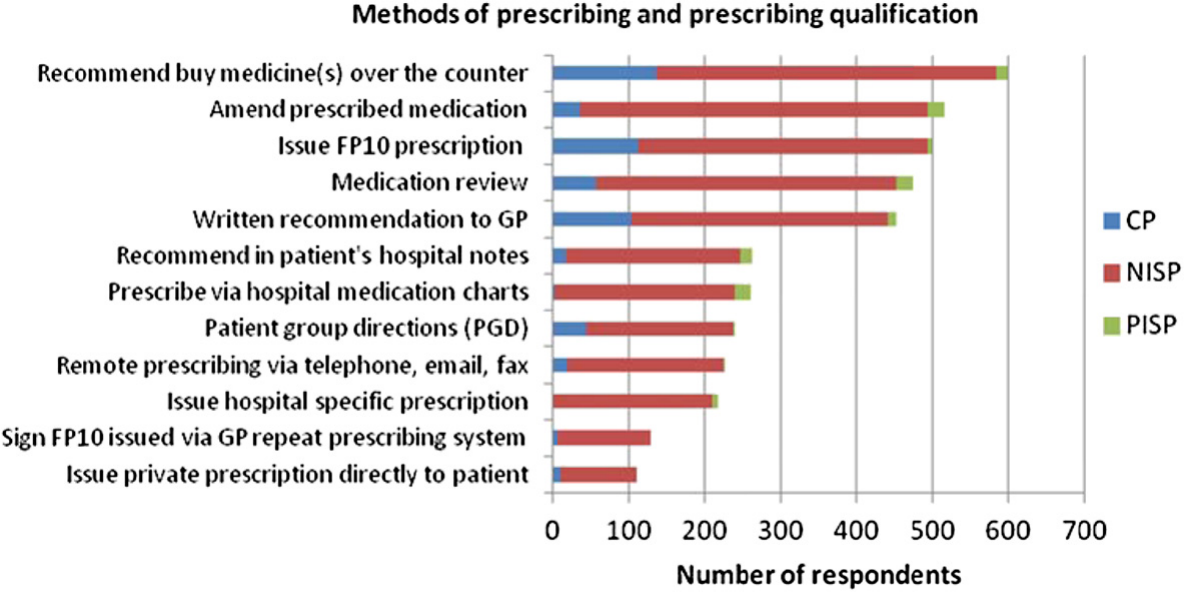
Methods of prescribing and prescribing qualification.

Participants reported that they used the prescribing qualification in a variety of ways (see Figure 4). The most common method cited was to make recommendations for patients to buy medicine(s) over the counter (n = 610, 80.6%). Over two thirds of community practitioners (n = 136, 68.7%) reported that they used it in this way. The most common method reported by NISPs (n = 458, 77.6%), and PISPs (n = 22, 62.9%) was to amend prescribed medication. Medication review was also reported to be conducted by a similar number of PISPs (n = 22, 62.9%) (see Figure 5).

**Figure 6:**
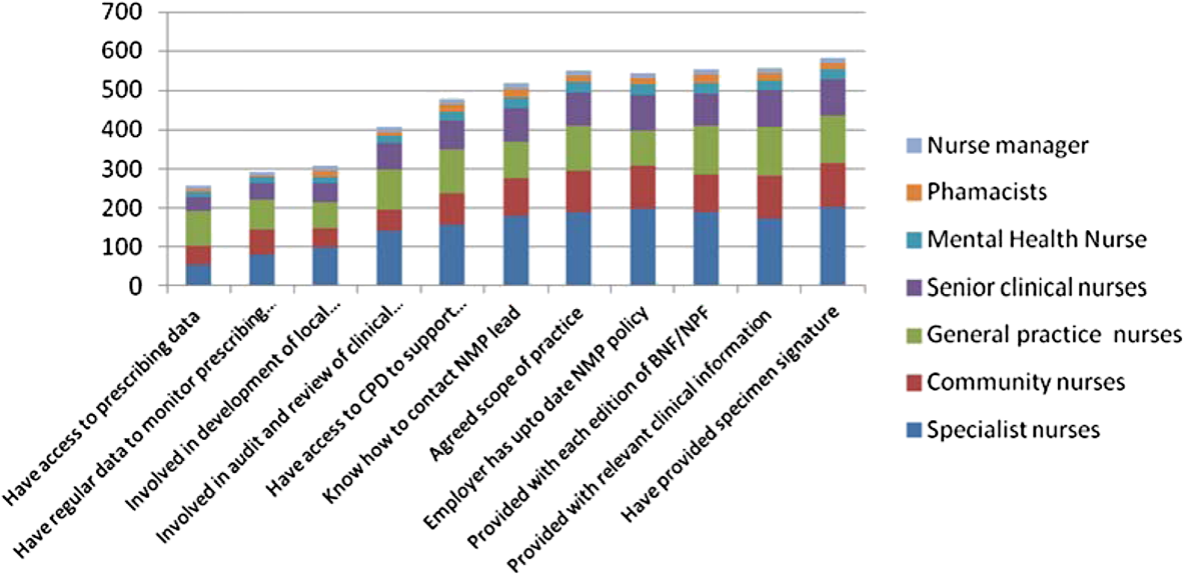
The extent to which to safety and clinical governance systems are in place and job title.

Using GLM it was evident that the number of ways the prescribing qualification was used was significantly affected by job title, employer, and care setting (p < 0.001). For example, a significantly greater number of general practice nurses, those employed in general practice, participants working in secondary care and those with more experience prior to undertaking the prescribing programme reported that they used the prescribing qualification in 6 or more ways (p < 0.001).

Of the community practitioners (n = 59) who reported they did not prescribe, 54% (n = 32) recommended over-the-counter (OTC) medicines to patients, and 42% (n = 25) recommended medications for general practitioners to prescribe for patients.

### Safety and clinical governance systems

Table 3 provides a summary of the extent to which participants reported that safety and clinical governance systems were in place. Over 90% of respondents reported that they had provided their employer with a specimen signature and received each edition of the British National Formulary (and/or the Nurse Prescribers Formulary for Community Practitioners). Only 328 (43.7%) reported that their employer provided them with regular data to monitor their prescribing practice, and only 281 (37.3%) were able to access their own prescribing data.

**Table 3:** The extent to which to safety and clinical governance systems are in place

***n = number of respondents who answered the question***	**Yes**		**No**	
	***n***	**%**	***n***	**%**
1. I have provided my employer with a specimen signature (n = 759)	694	91.4	65	8.6
2. My employer provides me with each edition of the BNF/the NPF for Community Practitioners (n = 714)	655	91.2	59	8.6
3. My employer ensures that I receive all relevant clinical information e.g. Patient Safety Notices, Drug Alerts and Hazard Warnings? (n = 758)	678	89.5	80	10.5
4. My employer has an up-to-date NMP policy (n = 740)	655	88.5	85	11.5
5. My scope of practice has been agreed with my employer (n = 754)	642	85.1	112	14.9
6. I know how to contact my NMP lead (n = 754)	629	83.4	125	16.6
7. I have access to CPD to support me in prescribing role (via employer/trust/independently) (n = 755)	561	74.3	194	25.7
8. I am involved with regular clinical audit and review of my clinical services (n = 750)	480	64.0	270	26.0
9. My employer has involved me in the development of local formularies and guidelines (n = 755)	358	47.7	397	52.6
10. My employer provides me with regular data to monitor my prescribing practice (n = 751)	328	43.7	423	16.3
11. I am able to access my own prescribing data (via PACT or otherwise) (n = 746)	281	37.3	465	62.3

(NMP = non medical prescribing, BNF = British National Formulary, NPF = Nurse Prescribers Formulary, PACT = Prescription analysis and cost trend, CPD = continuing professional development).

**Figure 7:**
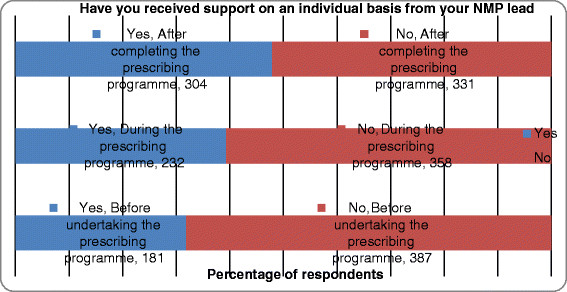
Support received from the non-medical prescribing lead on an individual basis.

Using GLM it was evident that the extent to which safety and clinical governance systems were in place was significantly affected by job title, employer, and care setting, and prescribing qualification. For example, a significantly greater number of specialist nurses, those employed in acute trusts, participants working in mental health and those with the NISP qualification reported 6 or more clinical governance systems were in place (p < 0.001). Significantly fewer clinical governance systems were reported by community nurses and those with the community practitioner qualification (see Figure 7).

### Support from NMP lead

The level of support participants received from their NMP lead before, during and after the prescribing programme is shown in Figure 7. A greater number of respondents (n = 304, 47.8%) reported that they received support after they had completed the prescribing programme. Using chi-square analysis it was evident that NISPs, those who worked in mental health, or had been qualified for less than a year received significantly greater levels of support at each of these three stages (p < 0.001). Significantly fewer community nurses, pharmacists, those employed by PCTs, primary care, and those qualified for more than 5 years reported that they had received any support from their NMP lead (p < 0.001).

## Discussion

This is the first study of NMP within one SHA which provides detailed information about the numbers and types of NMPs, their prescribing practice and clinical governance arrangements. It therefore provides an important overview of the development of NMP across a large geographical area of England.

There are some limitations with the data set, in that email addresses of NMPs were not provided by NMP leads representing employees of six PCTs (including community trusts and other provider services). We therefore acknowledge an under-representation of NMPs employed by PCTs, particularly in Hertfordshire and Bedfordshire. The ratio of NISPs to PISPS, AHPs and optometrists in our sample reflects national data on NMP [9]. The high numbers of NISPs is unsurprising given the large nursing workforce in England, plus the fact that prescribing rights were granted to nurses first. Our response rate is 2% lower than a recent national evaluation of nurse and pharmacist independent prescribing [24]. Given the similar demographic profile of our sample to previous national evaluations of NMPs [23,24], we are confident that our findings present an accurate picture of this population. However, the proportion of community practitioner prescribers in our sample is lower than expected and is probably due to shortfalls in data provided by PCTs. While there have been national surveys of NISPs and PIPs, there is a lack of similar data on community practitioners with which to compare. It should also be noted that the data is self-report data, and therefore information such as items prescribed per week, are likely to be an estimate.

### Demographic profile

In-line with previous national evidence [23,24,28], the majority of NMPs in this SHA were employed by PCTs and based in primary care. This reflects the organisation of the NHS in England and recent policy drives to provide care closer to home through services provided in the community [29]. Two thirds of PISPs were employed in secondary care; this is higher than reported by previously [24] where 36% were in secondary care and 55% in general practice. Overall a third of NMPs worked in secondary care; which is similar to that reported previously [23,24,28]. This indicates that NMP is developing in line with policy intention that it would contribute to improving access and quality of care in a range of settings [1].

Overall, the level of education and experience that NMPs had was equivalent to previous surveys [23,24,28]. Guidance specifies that applicants for the NMP programme must have at least one year’s experience in the area in which they intend to prescribe [1]. Although around 90% of our sample had this experience (and 59.1% had over 5 years’ experience), 10.5% did not. Importantly, those in our sample with more prior experience made greater use of the prescribing qualification and prescribed more frequently than those with lesser experience. This highlights that experience helps to maximise use of the NMP role. Similarly, while most respondents had undertaken specialist education in their area of practice prior to undertaking prescribing, 24.5% had not. It has been found that nurses who acquire prior specialist knowledge are more likely to report that the prescribing course met their learning needs and prepared them to prescribe [30,31]. Patients have also been reported to have greater confidence in nurses who have more experience and specialist knowledge in their area of practice [32,33]. This reinforces the need to ensure that those selected for prescribing training have acquired the necessary specialist knowledge and experience [4].

### Prescribing patterns

A lack of prescribing activity is considered wasteful in terms of the time and expenses incurred for training [34] and failure to deliver predicted service improvement. Therefore, it is important to understand why some qualified NMPs do not prescribe. Although over 90% of NISPs reported that they currently prescribed medicines, approximately a third of PISPS, AHPs, and community practitioner prescribers, indicated that they did not. Prescribing rates amongst different groups have varied considerably since NMP was first introduced, for example, district nurses prescribe more frequently than health visitors [35], and NISPs [36], more frequently than community practitioner prescribers [37], mental health nurses [38], or pharmacists [24]. Among those NMPs who were currently prescribing, similar differences in prescribing patterns were found in this study, with lower rates reported by community practitioners prescribers, mental health nurses and PISPs. There are multiple factors, as well as differences in roles and practice settings, known to influence prescribing practice [39-41]. This study provides further insight into factors affecting this variation.

The main reason given for not prescribing was that participants no longer worked in a role that required this activity. This provides some reassurance that some of the initial barriers to NMP (including restrictions at a local level such as lack of access to prescription pads and inability to generate electronic prescriptions) are now less problematic [23]. However, these problems continued to restrict use of the community practitioner prescribing qualification, perhaps reflecting the difficulty of accessing and using electronic patient records in general practice where different IT systems are in place.

Of those who did prescribe, the rate of independent prescribing by nurses was similar to that reported in 2006 by nurse independent prescribers [23]. Nurses employed in general practice, however, prescribed significantly more items than those of other employers. In addition to prescribing more frequently, nurses in general practice, treating patients with diabetes [30] and dermatology [42], are known to prescribe for a greater range of conditions. This perhaps reflects the broad range of conditions encountered by these nurses and so the greater opportunity to prescribe.

Prescribing rates were influenced by the level of support received from the NMP lead before, during and after prescribing training. Those with less support (i.e. PISPs and community practitioner prescribers) generally prescribed less frequently. Interestingly, the least number of items prescribed was by those employed by mental health nurses who actually received the highest level of support. This anomaly may indicate the presence of other factors that influence the rate of prescribing in mental health. A lack of support from clinicians, for example, has been cited [43] as a barrier to prescribing by this group however; further research exploring these barriers is required. Overall, levels of support were inconsistent, in-line with previous study findings [15]. That those qualified for less than a year received more support perhaps indicates an increase in governance arrangements to provide support to NMP in recent times.

Supplementary prescribing was used infrequently and mainly by a few participants confined to this mode of prescribing through their type of qualification, organisational policy, or restrictions on what medicines can be prescribing via independent prescribing. This contributes to growing national evidence on the low use of supplementary prescribing (23, 44). Given that the main purpose of NMP was to maximise access and improve service efficiency, the continued usefullness of SP is questionable. This should be borne in mind by those involved in developing guidance on the extension of prescribing rights for other professionals.

### Ways of using the prescribing qualification

Historically, the success of NMP has been measured by the numbers actively prescribing or the frequency of prescribing. While this is important, this is the first study to provide evidence that NMPs engage in a range of other activities that can also impact on service efficiency, quality of care and patient outcomes. Despite approximately a third of community practitioner prescribers and PISPs reporting they did not prescribe, 54% of the those community practitioners who were not prescribing recommended OTC medicines to patients, and 42% recommended medications for general practitioners to prescribe for patients. Furthermore, the majority of PISPS and NISPS amended prescribed medications, undertook medication reviews and made recommendations to general practitioners. Nurses employed in general practice and in acute trusts reported that they used the qualification in significantly more ways than other groups. An appreciation of these activities is necessary if NMPs are to be fully supported in their role. Further research designed to explore these activities is required if we are to fully understand the benefits (including cost benefits) of NMPs to service delivery. Crucially, if data on involvement in these medicines management activities is not captured then the true worth of NMP activity with respect to patient outcomes and the efficiency of care processes will not be recognised. This is of particular importance during the current economic climate and period of uncertainty regarding the re-organisation of the NHS.

### Governance issues

For the most part, clinical governance arrangements were reported to be working, with the exception of the ability to obtain prescribing data and monitor or audit prescribing activity. These activities are important as they can provide a useful focus for clinical review, demonstrate evidence of safety and efficiency and highlight areas for continuing professional development. That fewer governance systems were in place for community practitioner prescribers may reflect the difficulties of maintaining procedures in community settings where lack of IT infrastructure can hamper communication and support for those working peripatetically. Poor infrastructure, lack of confidence, and poor access to continuing professional development were factors reported to prevent this group from prescribing. These findings, along with previous research on NMP governance [15], provide support for the need to further develop the clinical governance systems within which NMPs work.

## Conclusion

NMP in this SHA reflects national development of this relatively new role in that the majority of NMPs are nurses based in primary care, with fewer pharmacist and AHP prescribers. In addition to prescribing, this workforce contributes to medicines management activities in a range of care settings. The extent, to which NMPs prescribed, was influenced by a number of factors including employer, the level of experience prior to becoming a NMP, and existence of governance procedures and support for the prescribing role. If NMPs are to maximise their contribution to patients and healthcare services robust governance and support from healthcare organisations is essential. This requirement will increase as the NMP workforce grows. The continued use of supplementary prescribing, which requires greater co-working with a doctor and is used less frequently than independent prescribing, as a first step towards prescribing rights for health professionals is questionable if maximum efficiency is sought. These are important points that need to be considered by those responsible for developing NMP in the UK and other countries around the world.

## Competing interests

The author(s) declare that they have no competing interests.

## Authors’ contribution

MC was responsible for the study conception and design. NC, KS, and MC developed the questionnaire; NC performed the data collection and analysis. All authors participated in the drafting of this manuscript and have approved the final manuscript.

## Pre-publication history

The pre-publication history for this paper can be accessed here:

http://www.biomedcentral.com/1472-6963/12/138/prepub

## Supplementary Material

Additional file 1 **General Information.** The questionnaire is aimed at non medical prescribers (NMPs). It should take you about 15 minutes to complete. Most questions require you to tick the box(s) that apply. If you make a mistake just tick the box you do require and it will change automatically. You can also scroll backward through the pages if you want to change a previous answer. Once you reach the end click on ‘finish’ and your answers will automatically be saved and sent to us. Click here for file

## References

[B1] DoH Improving patient’s access to medicines 2006 A guide to implementing nurse and pharmacist independent prescribing within the NHS in England, London: DH

[B2] Ball J Implementing Nurse Prescribing: An Updated Review of Current Practice Internationally 2009 International Council of Nurses, Geneva

[B3] Kroezen M Van Dijk L Groenewegen PP Francke AL Nurse prescribing of medicines in Western European and Aglo-Saxon, countries: a systematic review of the literature BMC Health Serv Res 2011 11 127 10.1186/1472-6963-11-27 PMC3141384 21619565

[B4] NMC Standards of proficiency for nurse and midwife prescribers 2006 NMC, London

[B5] DoH Patients to get quicker access to medicines (Press Release) 2001 DH, London

[B6] Home Office (HO) Nurse and pharmacist independent prescribing, ‘mixing of medicines’, possession authorities under patient group directions and personal exemption provisions for Schedule 4 Part II drugs. Home Office circular 009/2012 2012 HO, London

[B7] DoH Supplementary Prescribing 2005 DH, London

[B8] DoH Optometrists to get independent prescribing rights (Press Release) 2007 DoH, London

[B9] Culley F NMC and Prescribing 2011 19th October: ANP’s 13th National Conference and CPD event, London

[B10] NHS Information Centre 2011 http://www.ic.nhs.uk

[B11] DoH Proposals to introduce prescribing responsibilities for paramedics: stakeholder engagement. Consultation paper 2010 DoH, London

[B12] Stewart D George J Bond C Cunningham S Diack L McCaig D Exploring patients’ perspectives of pharmacist supplementary prescribing in Scotland Pharm World Sci 2008 30 892 897 10.1007/s11096-008-9248-x 18787976

[B13] Cooper R Anderson C Avery T Bissell P Guillaume L Hutchinson A Lymn J Murphy E Ratcliffe J Ward P Stakeholders’ views of UK nurse and pharmacist supplementary prescribing J Health Res Policy 2008 13 4 215 221 10.1258/jhsrp.2008.008004 18806179

[B14] Brooks N Otway C Rashid C The patient’s view: the benefits and limitations of nurse prescribing Br J Community Nurs 2001 10.12968/bjcn.2001.6.7.7066 11865224

[B15] Courtenay M Carey N Stenner K Non-medical prescribing leads views on their role and the implementation of non-medical prescribing from a multi-organisational perspective BMC Health Serv Res 2011 11 142 10.1186/1472-6963-11-142 21635744 PMC3120647

[B16] Hobson R Scott J Sutton J Pharmacists and nurses as independent prescribers: exploring the patient’s perspective Fam Pract 2010 27 110 120 10.1093/fampra/cmp070 19858124

[B17] Courtenay M Carey N Stenner K Nurse prescriber-patient consultations: a case study in dermatology J Adv Nurs 2009 65 6 1207 1217 10.1111/j.1365-2648.2009.04974.x 19374682

[B18] Stenner K Courtenay M Benefits of nurse prescribing for patients in pain: nurse’s views J Adv Nurs 2008 63 1 27 35 10.1111/j.1365-2648.2008.04644.x 18503536

[B19] George J McCaig D Bond CM Cunningham ITS Diack HL Watson AM Stewart DC Supplementary prescribing: early experiences of pharmacists in Great Britain Ann Pharmacother 2006 40 1843 1850 10.1345/aph.1H227 16968824

[B20] Jones M Bennett J Lucas B Mental health nurse supplementary prescribing: experiences of mental health nurses, psychiatrists and patients J Adv Nurs 2007 59 488 496 10.1111/j.1365-2648.2007.04332.x 17681079

[B21] NPC A quick guide for commissioner 2010 NPC, London

[B22] Courtenay M Carey N Nurse Independent Prescribing and Nurse Supplementary Prescribing: Findings from a national questionnaire survey J Adv Nurs 2008 61 4 403 412 10.1111/j.1365-2648.2007.04534.x 18234038

[B23] Latter S Blenkinsopp A Smith A Evaluation of nurse and pharmacist independent prescribing 2010 DoH report, University of Southampton and Keele University

[B24] Latter S Courtenay M Effectiveness of nurse prescribing: a review of the literature J Clin Nurs 2004 13 26 32 10.1046/j.1365-2702.2003.00839.x 14687290

[B25] Courtenay M Carey N Stenner K A national evaluation of nurse prescribing in dermatology 2010 Unpublished, University of Surrey

[B26] Hill T Lewicki P STATISTICS Methods and Applications 2007 Statsoft, Tulsa, OK

[B27] Courtenay M Gordon J A survey of therapy areas in which nurses prescribe and CPD needs Nurse Prescribing 2009 7 6 255 262

[B28] DoH Shifting Care Closer to Home 2007 DoH, London

[B29] Courtenay M Carey NJ Preparing nurses to prescribe medicines for patients with diabetes: a national survey J Adv Nurs 2008 61 3 291 299 10.1111/j.1365-2648.2007.04512.x 18234038

[B30] Courtenay M Carey N Preparing nurses to prescribe medicines for patients with dermatological conditions J Adv Nurs 2006 55 6 698 707 10.1111/j.1365-2648.2006.03960.x 16925618

[B31] Courtenay M Stenner K Carey N The views of patients with diabetes about nurse prescribing Diabet Med 2010 27 1049 1054 20722679 10.1111/j.1464-5491.2010.03051.x

[B32] Courtenay M Carey N Stenner K Lawton S Peters J Patients views on nurse prescribing: effects on care, concordance and medicine taking Br J Dermatol 2010 164 2 396 401 21054336 10.1111/j.1365-2133.2010.10119.x

[B33] Bissell P Cooper R Guillaime L Anderson C Avery A Hutchinson A James V Lymn J Marsden E Murphy E Ratclife J Ward P Woolsey I An evaluation of supplementary prescribing in nursing and pharmacy 2008 Final report for the doH, University of Sheffield

[B34] Luker K McHugh GA Nurse prescribing from the community nurse’s perspective Int J Pharm Pract 2002 10 273 280 10.1211/096176702776868433

[B35] Latter S Maben J Myall M Courtenay M Young A Dunn N An Evaluation of Extended Formulary Independent Nurse Prescribing. Final Report 2005 Policy Research Programme Department of Health & University of Southampton, UK

[B36] While AE Biggs KSM Benefits and challenges of nurse prescribing J Adv Nurs 2004 45 6 559 567 10.1046/j.1365-2648.2003.02948.x 15012633

[B37] Dobel-Ober N Brimblecombe N Bradley E Nurse prescribing in mental health:national survey J Psychiatr Ment Health Nurs 2010 17 487 493 10.1111/j.1365-2850.2009.01541.x 20633075

[B38] Carey N Courtenay M An exploration of the continuing professional development needs of nurse independent prescribers and nurse supplementary prescribers J Clin Nurs 2010 19 208 216 10.1111/j.1365-2702.2009.02943.x 20500258

[B39] Bradley E Wain P Nolan P Putting mental health nurse prescribing into practice Nurse Prescribing 2008 6 1 15 19

[B40] Hall J Cantrill J Noyce P Professional issues. The information sources used by community nurse prescribers. Br J Nurs 2003 12 13 810 818 12920459 10.12968/bjon.2003.12.13.11349

[B41] Courtenay M Carey N Independent extended nurse prescribing for patients with skin conditions: a national questionnaire survey J Clin Nurs 2007 16 1247 1255 10.1111/j.1365-2702.2007.01788.x 17584342

[B42] Pate MX Robson D Rance J Ramirez NM Memon TC Bressington D Gray R Attitudes regarding mental health nurse prescribing among psychiatrists: a cross-sectional questionnaire study Int J Nurs Stud 2009 46 1467 1474 10.1016/j.ijnurstu.2009.04.010 19482282

[B43] Stenner K Courtenay M Cannons K Nurse prescribing for inpatient pain in the United Kingdom: a national questionnaire survey Int J Nurs Stud 2011 10.1016/j.ijnurstu.2011.01.009 21316672

