# Supplementary material for: An overiew of non medical prescribing across one strategic health authority: a questionnaire survey
Source: BMC Health Serv Res. 2012 Jun 1;12:138. doi: 10.1186/1472-6963-12-138 (PMC3420322; doi:10.1186/1472-6963-12-138)
Supplement: Additional file 1 — General Information. The questionnaire is aimed at non medical prescribers (NMPs). It should take you about 15 minutes to complete. Most questions require you to tick the box(s) that apply. If you make a mistake just tick the box you do require and it will change automatically. You can also scroll backward through the pages if you want to change a previous answer. Once you reach the end click on ‘finish’ and your answers will automatically be saved and sent to us. [file 1472-6963-12-138-S1.pdf]

1. General Information

The questionnaire is aimed at non medical prescribers (NMPs). It should take you about 15 minutes to complete. Most questions require you to tick the box(s) that apply. If you make a mistake just tick the box you do require and it will change automatically. You can also scroll backward through the pages if you want to change a previous answer. Once you reach the end click on 'finish' and your answers will automatically be saved and sent to us.

1. Job Title

2. What is your job band?

- 5
- 6
- 7
- 8a
- 8b
- 8c
- 8d
- Other (please specify)

3. What geographical area(s) do you cover? (Please tick all that apply)

- Bedfordshire
- Cambridgeshire
- Essex
- Hertfordshire
- Norfolk
- Suffolk

4. Who is your employer? (Please tick all that apply)

- Acute Trust
- General Practice
- Mental Health
- PCT
- Other (please specify)

5. How many hours do you currently work?

- Full time
- Part-time, 21 hours or more per week
- Part time, between 10-20 hours per week
- Part-time, up to 10 hours per week

6. What age range are you in?

- 25 or under
- 26-35
- 36-45
- 46-55
- 56 or over

7. What is the highest level of education/academic qualification you have obtained?

- Certificate
- Diploma
- Degree
- Masters
- PhD

8. Which care setting do you work in?

- Primary
- Secondary
- Primary & Secondary
- Intermediate
- Tertiary
- Mental Health
- Other (please specify)

9. What type of service (s) do you provide? (please tick all that apply)

- ☐ NHS hospital in-patient
- ☐ NHS hospital out-patient
- ☐ NHS community clinic
- ☐ Out of hours
- ☐ Walk in centre
- ☐ General Practice service
- ☐ Community
- ☐ Independent Sector
- ☐ HM Prison Services
- ☐ Other (please specify)

10. How many qualified NMPs are there in your team/work unit?

○ 1	○ 2	○ 3
○ 4	○ 5	○ 6
○ 7	○ 8	○ 9
○ 10 or more	○ unsure	

11. Please answer the following questions about the number of NMPs in your area of practice

	Yes	No	Don't know
a)Do you think there is a need for more NMPs in your team?	○	○	○
b)Are there plans to increase the number of NMPs in your team?	○	○	○
c)Are any members of your team currently on the NMP course?	○	○	○
d)Is there anything that hampers expanding the numbers of NMPs in your team?	○	○	○

If YES plese specify

2. Prescribing Background

1. What prescribing qualification do you have?

- ☐ Community Practitioner Nurse Prescriber (v100)
- ☐ Community Practitoner Prescriber without a Specialist Practitioner Qualification (v150)
- ☐ Nurse Independent/Supplementary Prescriber (v200 & v300)
- ☐ Pharmacist Supplementary Prescriber
- ☐ Pharmacist Independent/Supplementary Prescriber
- ☐ Physiotherapist Supplementary Prescriber
- ☐ Podiatrist/chiropodist Supplementary Prescriber
- ☐ Radiographer Supplementary Prescriber
- ☐ Optometrist Supplementary Prescriber
- ☐ Optometrist Independent/Supplementary Prescriber

2. How many years have you been qualified as a prescriber?

- ○ <1 year
- ○ 1-3 years
- ○ 3-5 years
- ○ >5 years

3. How much experience did you have in your main area of prescribing practice before undertaking the prescribing programme?

- ○ < 1year
- ○ 1-2 years
- ○ 2-5 years
- ○ >5 years

4. Prior to undertaking the prescribing programme, had you undergone any specialist training in your area of prescribing practice? (please tick all that apply)

- ☐ Diploma level module/s
- ☐ Degree level module/s
- ☐ Masters level module/s
- ☐ Accredited study day/s (e.g. University/RCN)
- ☐ No specialist training
- ☐ Other training (e.g. drug company study days/ conferences) (please specify)

5. Please indicate what training experience you had in the following areas BEFORE undertaking the prescribing programme (please tick all that apply)

	a)Accredited learning through an institution (e.g. university, professional body or prescribing forum)	b) Learning not accredited via university (e.g. drug company training day, conferences, independent provider)	c) Experiential learning/ on the job training	d) None
a) Assessment & diagnostic skills	☐	☐	☐	☐
b) Pharmacology	☐	☐	☐	☐
c) Numeracy	☐	☐	☐	☐

6. Please indicate what training experience you have had in the following areas SINCE qualifying as a prescriber (please tick all that apply)

	a) Accredited learning through an institution (e.g. university, professional body or prescribing forum)	b) Learning not accredited via university (e.g. drug company training day, conferences, independent provider)	c) Experiential learning/ on the job training	d) None
a) Assessment & Diagnostic skills	☐	☐	☐	☐
b) Pharmacology	☐	☐	☐	☐
c) Numeracy	☐	☐	☐	☐

3. Prescribing Practice

1. Which method of prescribing do you currently use? (please tick all that apply)

- [ ] Independent Prescribing (IP)
- [ ] Supplementary Prescribing (SP-using a clinical management plan)
- [ ] I do not prescribe (please give reason(s) below) (if you don't prescribe please skip until Q4)

2. If you use supplementary prescribing please indicate the reason(s)why: (please tick all that apply)
(if you don't use SP please skip to the next question)

- [ ] a)Only qualified as supplementary prescriber
- [ ] b)Current formulary restrictions on controlled drugs
- [ ] c)Trust policy
- [ ] d)Personal preference
- [ ] e) Other (please specify)

3. Please indicate how many items you currently prescribe in a typical week using IP and/or SP:
(please put a tick in both rows)

	0	1-5	6-10	11-20	21-30	31-40	41-50	>50
IP	○	○	○	○	○	○	○	○
SP	○	○	○	○	○	○	○	○

4. Please indicate how often you use your prescribing qualification in the following ways:

	never	rarely (once a month or less)	infrequently (every 2-3 weeks)	regularly (weekly)	frequently (2-3 times a week)	very frequently (daily)
Remote prescribing via telephone, email, fax etc.	○	○	○	○	○	○
Make recommendation via letter/email or telephone to GP for medicine(s) to be prescribed	○	○	○	○	○	○
Make recommendation via patient's hospital notes for medicine(s) to be prescribed	○	○	○	○	○	○
Make recommendation for patient to buy medicine(s) over the counter	○	○	○	○	○	○
Medication review	○	○	○	○	○	○
Amend prescribed medication (i.e. stop, alter or correct dosage)	○	○	○	○	○	○
Issue FP10 prescription directly to patient	○	○	○	○	○	○
Sign FP10 prescriptions printed via GP repeat prescribing system	○	○	○	○	○	○
Issue hospital specific prescription directly to patient	○	○	○	○	○	○
Issue private prescription directly to patient	○	○	○	○	○	○
Prescribe via hospital medication charts	○	○	○	○	○	○
Supply and administer medicines via Patient group directions (PGD)	○	○	○	○	○	○
other	○	○	○	○	○	○

please specify in what other ways you use your prescribing qualification

5. In which therapy areas do you prescribe (please tick all that apply)?
(if you DON'T prescribe please skip to the next question)

- ☐ Anaesthesia
- ☐ Anticoagulation
- ☐ Cardiovascular
- ☐ Dementia
- ☐ Dermatology
- ☐ Diabetes
- ☐ Drug & substance Misuse
- ☐ Emergency medicine (A/E)
- ☐ Family planning
- ☐ Gastroenterology
- ☐ Haematology
- ☐ Hepatology
- ☐ Immunisations & Vaccinations
- ☐ Mental health
- ☐ Minor ailments
- ☐ Neurology
- ☐ Oncology
- ☐ Pain
- ☐ Palliative care
- ☐ Renal
- ☐ Respiratory
- ☐ Rheumatology
- ☐ Sexual Health
- ☐ Smoking cessation
- ☐ Urology
- ☐ Womens health
- ☐ Wound care

Other (please specify)

4. Governance and Support

1. Please indicate your experience in the following areas:(Please tick each row)

	Yes	No
My employer provides me with each edition of the BNF/NPF	☐	☐
My employer has an up-to-date non medical prescribing policy	☐	☐
My employer ensures that I receive all relevant clinical information e.g. patient safety notices & drug alerts	☐	☐
My employer has involved me in the development of local formularies and guidelines	☐	☐
I have provided my employer with a specimen signature	☐	☐
My scope of prescribing practice has been agreed with my employer	☐	☐
My employer provides me with regular data to monitor my prescribing practice	☐	☐
I am able to monitor/ access my own prescribing data (via PACT or otherwise)	☐	☐
I have access to appropriate CPD to support me in my prescribing role (via employer/trust or independently)	☐	☐
I am involved with regular clinical audit and review of my clinical services	☐	☐
I know how to contact my NMP lead	☐	☐

2. Have you received any support on an individual basis from your NMP lead? (Please tick each row)

	Yes	No	Not required
a) Before undertaking the prescribing programme	○	○	○
b) During the prescribing programme	○	○	○
c) After completing the prescribing programme	○	○	○

3. The statements below are designed to explore your experiences of your PREPARATION for the prescribing role: (please rate each statement)

	strongly disagree	disagree	neither agree or disagree	agree	strongly agree
a)My employer was supportive of my request to undertake the prescribing programme					
b)I had difficulties in identifying a suitable designated medical practitioner (DMP) for the prescribing programme					
c)I had a clear idea of what would be expected from me during the course					
d)I agreed my prescribing role with my employer prior to undertaking the prescribing programme					

4. The statements below are designed to explore your experiences DURING the prescribing programme: (please rate each statement)

	strongly disagree	disagree	neither agree or disagree	agree	strongly agree
a)I was able to access the support I required from my DMP					
b)My employer was supportive of programme requirements (e.g. study leave for taught element and 12 days of clinical practice)					
c)My DMP was able to provide me with appropriate learning opportunities					
d) The programme equipped me with the appropriate knowledge, skills and competencies to prescribe in my area of clinical practice					
e)I am satisfied with the level of support and supervision I received from my DMP					
f)I had difficulty meeting the learning outcomes of the programme					

5. If you experienced difficulties completing the learning outcomes of the prescribing programme please indicate what they were: (please tick all that apply) (If you did not experience any difficulties please skip to the next question)

- ☐ a) Assessment & Diagnostic skills
- ☐ b) Pharmacology
- ☐ c) Numeracy
- ☐ d) Time/volume of study
- ☐ e) level of academic study
- ☐ Other (please specify)

6. The statements below are designed to explore your experiences AFTER the prescribing programme: (please rate each statement)

	strongly disagree	disagree	neither agree or disagree	agree	strongly agree	N/A
I continue to receive support from my DMP/other clinician for my prescribing role	○	○	○	○	○	○
Prescribing ensures better use of my skills	○	○	○	○	○	○
My employer provides an appropriate level of support for my prescribing role (e.g. prescribing forum, action learning sets)	○	○	○	○	○	○
The ability to prescribe improves the quality of care I am able to offer patients	○	○	○	○	○	○
My peers/ team members are supportive of my prescribing role	○	○	○	○	○	○
Opposition or resistance (from doctors, and other health care professionals) has hampered my ability to use my prescribing qualification	○	○	○	○	○	○
I receive support for my prescribing role from the local, or trust, pharmacist	○	○	○	○	○	○
I am able to prescribe all the drugs I need in order to do my job (if disagree or strongly disagree please provide details below)	○	○	○	○	○	○
Overall I am satisfied with the level of support I receive in my role as a prescriber	○	○	○	○	○	○
I am limited in my prescribing practice (if agree or strongly agree please provide details below)	○	○	○	○	○	○

Please provide any free text comments here

5. Thank you

Thank you for taking the time to complete this survey

1. Please indicate if you are interested in receiving a summary of the results of this survey, or would be interested in being considered for invitation to take part in the next phase of this research.

- ☐ Yes, I would like a summary of the survey results
- ☐ Yes, I may be interested in taking part in further research

2. If you answered yes to either of the above, please provide contact details. Your details will remain confidential and will not be published in the survey results.

Name:

Employer:

Address:

Address 2:

City/Town:

Postcode:

Email Address:

Phone Number:

3. General comments
